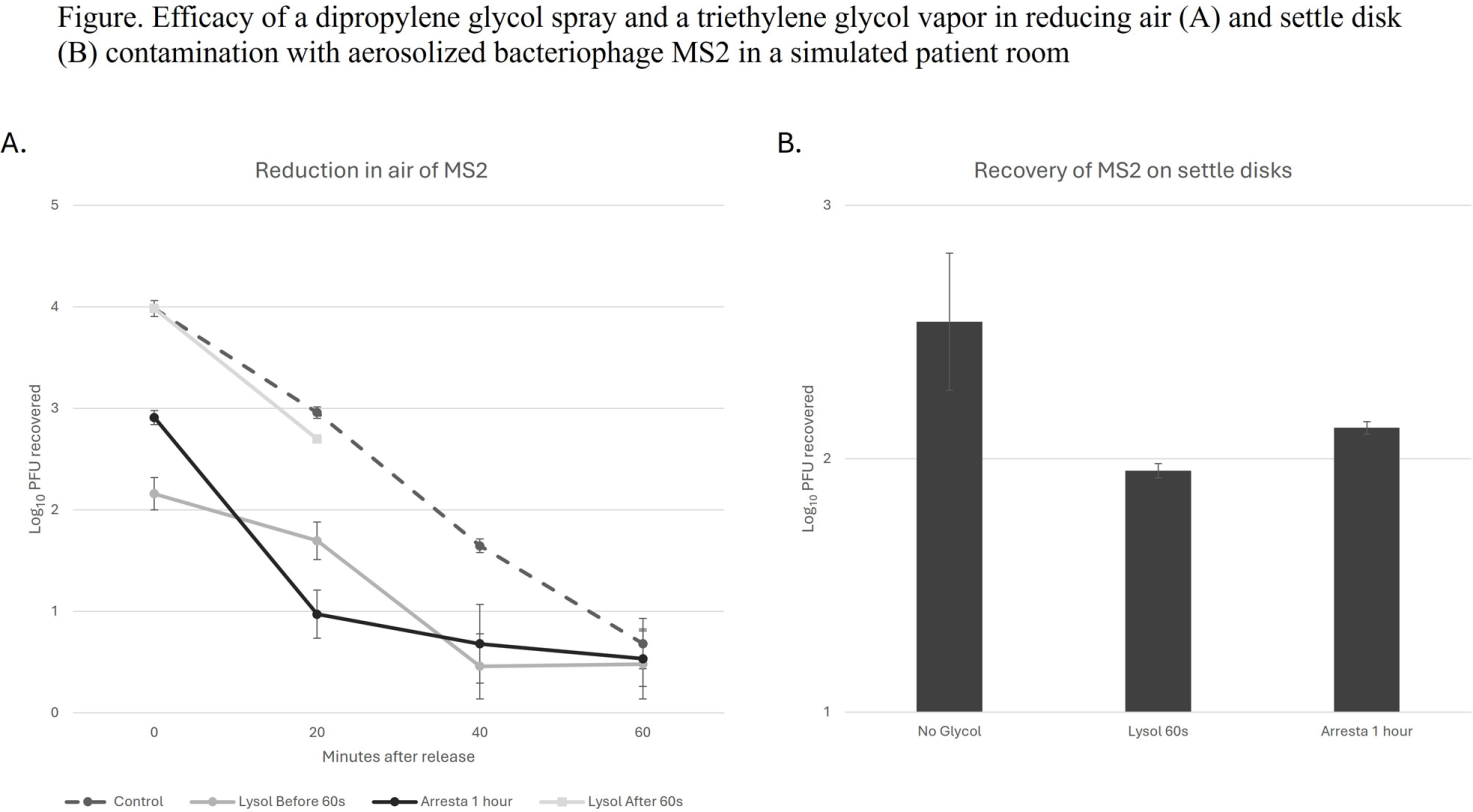# 217 Acinetobacter spp. Infections Were Commonly Healthcare-Associated but Not Hospital-Onset, Denver Safety-Net Health System, 2015–2025

**DOI:** 10.1017/ash.2026.10600

**Published:** 2026-06-23

**Authors:** Jennifer Cadnum, Samir Memic, Maria Torres-Teran, Amelia Milner, Curtis Donskey

**Affiliations:** 1 Cleveland VA Medical Center; 2 VA Medical Center

## Abstract

**Background:** Daily toothbrushing is recommended for all hospitalized patients and long-term care facility (LTCF) residents because it has been associated with significantly lower rates of healthcare-associated pneumonia. However, limited information is available on effective strategies to improve education of patients on oral hygiene. **Methods:** We surveyed a convenience sample of 60 patients hospitalized on medical/surgical or rehabilitation wards at a Veterans Affairs medical center regarding oral hygiene education and practices. Medical records were reviewed to determine if education on oral hygiene was documented by nursing staff as is recommended by facility protocols. Based on the initial survey results, we piloted an intervention in which educational posters were placed beside the mirror in patient bathrooms and small educational placards were placed on meal trays. Patients were surveyed to determine the impact of the intervention. **Results:** Of the 60 patients completing the initial survey, 55 (92%) practiced oral hygiene at home, and 19 (32%) had partial or complete dentures. Nursing notes documented oral hygiene education for 39 (65%) patients, but only 18 (30%) recalled receiving education, and none were aware that oral hygiene reduces the risk for pneumonia. After the intervention, 35% of patients were aware of the sign by the sink, 62% were aware of the reminder on meal trays, and 35% were aware that oral hygiene reduces the risk for pneumonia (Figure). **Conclusions:** Simple visual educational tools can improve awareness of oral hygiene and its role in reducing the risk for hospital-acquired pneumonia. Studies are needed to determine if interventions using these tools will improve oral hygiene practices by patients.

**Figure:**